# A population-based survey of the prevalence of self-reported acute gastrointestinal illness in Zhejiang Province, China

**DOI:** 10.1371/journal.pone.0268717

**Published:** 2022-05-18

**Authors:** Ji-Kai Wang, Yue He, Li-Li Chen, He-Xiang Zhang, Xiao-Juan Qi, Liang Sun, Shuang-Feng Zhang, Jiang Chen, Rong-Hua Zhang

**Affiliations:** Department of Nutrition and Food Safety, Zhejiang Provincial Center for Disease Control and Prevention, Hangzhou, China; University of Patras, GREECE

## Abstract

Acute gastrointestinal illness (AGI) is a prevalent public health concern worldwide. This study investigated the magnitude, distribution and burden of self-reported AGI among residents of Zhejiang Province, China. A face-to-face household survey was conducted using a multi-stage stratified random sampling method in 10 counties in Zhejiang Province between July 2018 and June 2019. In total, 12,021 participants were recruited. The prevalence of AGI 28 days after standardization was 1.8% (95% confidence interval (CI), 1.6–2.1), with an incidence rate of 0.24 episodes of AGI per person-year and an estimated 14 million cases of AGI in Zhejiang Province. Univariate and multivariable analyses showed a higher AGI prevalence among people who performed housework and were unemployed in summer and autumn among respondents living in western or northern cities (*p* < 0.05). More than 50% of AGI cases were attributed to the consumption of contaminated food. The disease burden caused by AGI in Zhejiang Province was approximately 975 million Chinses yuan (CNY). These results indicated that the disease burden of AGI in Zhejiang Province should be addressed and highlights the need for an improved active surveillance system of foodborne diseases to assess the impact of AGI on society and health.

## 1. Introduction

Acute gastrointestinal illness (AGI) results from various causes and factors. The signs and symptoms include diarrhea, vomiting, nausea, fever, and other systemic symptoms [1]. AGI is an important cause of morbidity and mortality in all age groups [2]. Foodborne diseases (FBDs) represent a vital public health issue worldwide, with an estimated 600 million (almost 1 in 10) people affected and 420,000 deaths annually due to FBDs [3,4]. FBDs generally occur due to infections by bacteria, fungi, viruses, etiological agents, parasites, and intestinal parasites, as well as prions and contaminants derived from the environment, operators, storage, and transport [5,6]. In FBDs, AGI is a classic symptom that can lead to diarrhea or vomiting [7]. Diarrhea occurrence is common, causing a wide spectrum of signs and symptoms ranging from minor discomfort to dehydration, which may result in death. Globally, an estimated 1.31 million people of all ages die of diarrhea [8]. In both developed countries and low-income regions worldwide, AGI is a major cause of morbidity and mortality [9–11]. A retrospective Canadian study that applied an AGI survey reported an estimated 0.57 episodes per person-year and almost 19.5 million episodes [12]. Similarly, in Sweden, the overall incidence rate was 360/1000 persons per year [13]. Moreover, AGI imposes a substantial economic burden on the population and health care system [14]. Therefore, there is an urgent need to estimate the prevalence of AGI in the population and understand the economic burden.

Many episodes of AGI are captured by traditional data from hospital and laboratory surveillance, which excludes data from health care systems and communities [15]. Thus, the prevalence of AGI cannot be estimated accurately by traditional surveillance owing to the large gaps in underreported AGI collection, etiology detection, and laboratory capacity [16,17]. In this context, more effective and intuitive surveillance methods were adopted for FBDs and AGI. Previously, the China National Center for Food Safety Risk Assessment conducted population-based surveys to determine the burden and distribution of AGI in China [11,18], including 12-month, retrospective face-to-face surveys conducted between 2012 and 2013 in Gansu Province, Northwest China [19]. Although surveys of AGI are quite common, the actual number of cases differs from the reported number; thus, the health care system cannot fully capture the actual burden of AGI [20]. Accordingly, there is great importance in controlling the prevalence of AGI by developing health policy. Zhejiang Province, on the eastern coast and the most developed province in China, has different environmental and climate situations, as well as social and economic conditions. Therefore, the objective of the present study was to estimate the prevalence, magnitude, and distribution of self-reported AGI in Zhejiang Province, China.

## 2. Methods

### 2.1 Study design and site selection

A population-based survey of the prevalence of self-reported AGI was administered over a 12-month period in Zhejiang Province between July 2018 and June 2019, using a multi-stage stratified random sampling method. The selection of sentinel sites was based on geographical location, economic level, population density, and dietary habits. The sentinel sites were: (i) Jinhua City (5.36 million people and 4.75 million households in Jinhua, including 0.80 million people in Dongyang City and 0.76 million people in Wucheng District), (ii) Wenzhou City (9.12 million people and 2.92 million households in Wenzhou, including 0.75 million people in Longwan District and 0.76 million people in Pingyang County), (iii) Quzhou City (2.12 million people and 0.77 million households in Quzhou, including 0.46 million people in Kecheng District and 0.25 million people in Kaihua County), (iv) Jiaxing City (4.50 million people and 1.45 million households in Jiaxing, including 0.59 million people in Xiuzhou District and 0.82 million people in Tongxiang City), (v) Zhoushan City (1.12 million people and 0.43 million households in Zhoushan, including 0.38 million people in Putuo District and 0.08 million people in Shengsi County). The Ethics Review Committee of the Zhejiang Provincial Center for Disease Control and Prevention approved this study, and informed written consent was obtained.

### 2.2 Sample size determination and sampling procedures

The sample size was determined based on the resident population, the expected monthly prevalence rate, and the relative allowable error; an estimation (n = μ_α_^2^×π(1−π)/δ^2^) formula with an assumption of μ_α_ and 95% confidence interval of 1.96, a δ degree of error of 0.09π (0.45% allowable error), and an estimated prevalence of diarrhea in this population of 5% were applied. Thus, the required sample size was 9,011 individuals. To account for loss to follow-up, we increased the sample size to 12,000 across five cities, corresponding to at least 200 people in each city every month.

**Fig 1:**
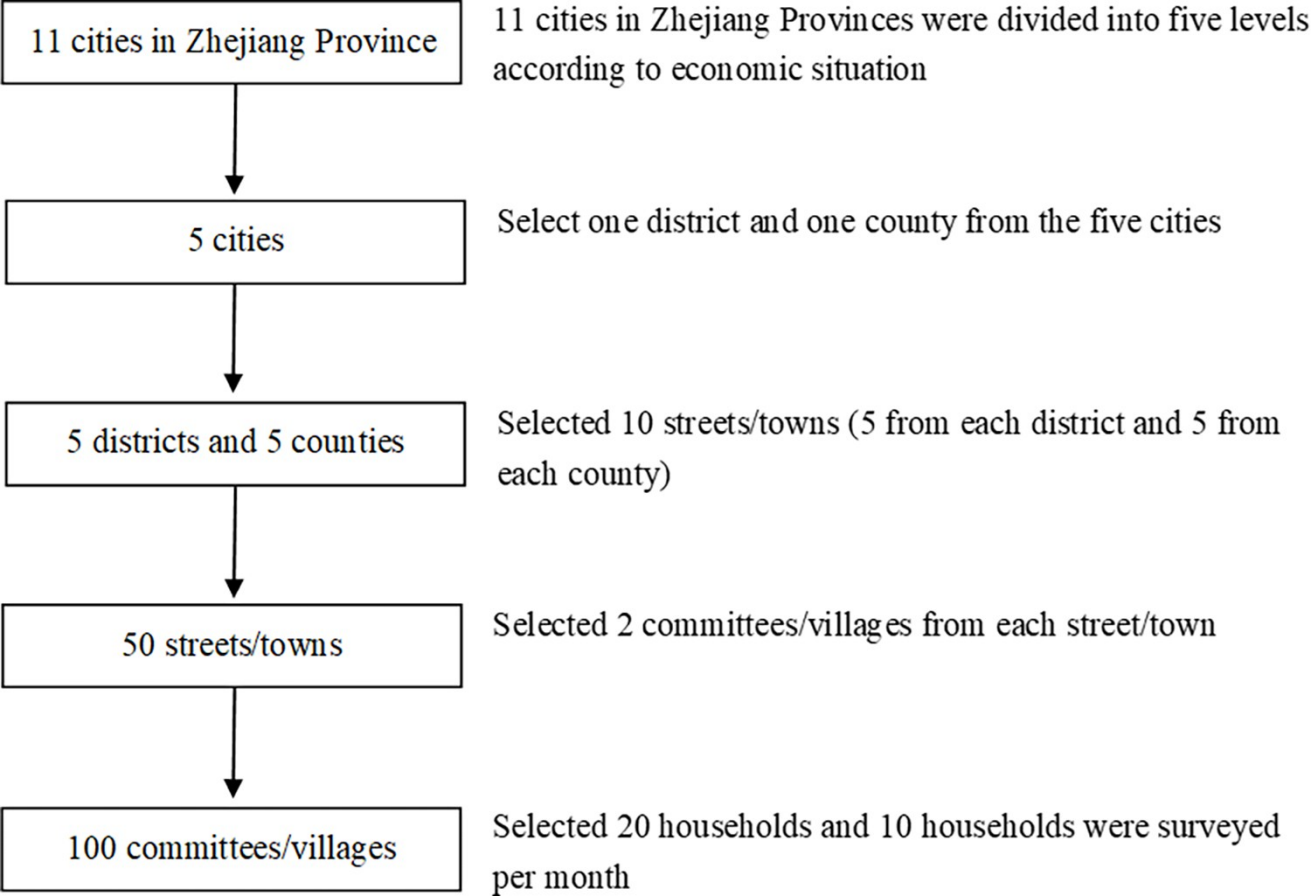
Flowchart of the sampling method.

A multi-stage sampling method was employed. First, 11 cities in Zhejiang Province were divided into five levels according to their economic status, and one city was selected from each level by a simple random sampling method. Second, simple random sampling was used to select one district and one county from each of the five cities. If there were fewer than 10 streets/towns in the municipal district or county of the city, the adjacent districts/counties were combined as a new district/county. In the third stage, in the selected municipal districts and counties, 10 streets/towns were selected (five from each district and five from each county) by simple random sampling. Fourth, we selected two committees/villages from each street/town by simple random sampling. If there were fewer than 200 households, neighboring committees/villages were merged. Finally, 120 households were selected by simple random sampling and 10 households were surveyed per month. The sampling framework is illustrated in **Fig 1**.

### 2.3 Data collection

The surveys were conducted by well-trained medical staff using a validated questionnaire. All participants were asked if they had experienced diarrhea or vomiting 28 days before the survey. If the participants responded yes to this question, we collected relevant information on their disease signs, timing, suspected reasons for the illness, travel history, treatment, hospital admission, whether a fecal sample was sent for clinical diagnosis, the social and economic impact of AGI, and the occurrence of AGI in other household members. We also asked about demographic characteristics, including the respondent’s sex, age, education, occupation, household size, type, and residence (see **Supporting information** for the translated study questionnaire used in this survey).

### 2.4 AGI case and operational definition

The definition of AGI from the Working Group on Abdominal Problems (WGAP) of the European Society of Intensive Care Medicine (ESICM) includes vomiting (emesis) at least once with gastrointestinal symptoms or diarrhea three or more times per day [21]. In this study, individuals were considered to have AGI if they met one or both of the following symptoms: (1) diarrhea, defined as three or more loose stools within 24 h, accompanied by abnormal fecal characteristics; and (2) vomiting (accompanied by content). Individuals with (1) intestinal cancer, inflammatory bowel diseases such as Crohn’s disease and ulcerative colitis, acute pancreatitis, irritable bowel syndrome, colitis, diverticulitis, or (2) pregnancy, excessive alcohol consumption, chemotherapy/radiotherapy, drugs, menstruation, food allergies, or some specific causes were excluded [22]. If the respondent reported multiple episodes (symptoms occurring more than twice in a 7-day period), we used only the latest episode.

### 2.5 Data analysis

EpiData version 3.1 (EpiData Association, Denmark) was used for data entry at each sentinel site. All the data were analyzed using IBM SPSS Statistics for Windows, version 22.0 (IBM Corp., Armonk, NY, USA). Yearly household income was classified as 0–19999 Chinese yuan (CNY), 20000–50000 CNY, and above 50000 CNY. The seasons were defined as winter (December to February), spring (March to May), summer (June to August), and autumn (September to November).

The response rate was calculated by dividing the number of completed surveys by the number of households visited. The monthly prevalence of AGI was calculated as the number of reported individuals with AGI in the 4 weeks prior to the face-to-face interview divided by the total number of respondents. The point prevalence of AGI was calculated as the proportion of cases with symptoms on the day of the interview. For the incidence rate calculations, respondents reporting multiple episodes were counted as a single episode. The incidence rate of AGI per person-year was determined according to the terms and formulas in Modern Epidemiology [23,24].

These results were adjusted for known differences between the investigated persons and the target population by weighting for age, sex and residence based on the data from the sixth nationwide census in Zhejiang Province. Chi square tests were used to verify the relationship between demographic characteristics and the prevalence of AGI. The mean duration of diarrhea according to different age groups was compared by analysis of variance (ANOVA). Statistical significance was set at *p* < 0.05. We estimated the odds ratios (ORs) for each of the demographic characteristics by univariate logistic regression and included those that were statistically significant in the multivariable logistic regression models. The variables in the logistic regression were not weighted because we aimed to obtain the relative ORs among the risk factors. The multivariable analysis applied the forward selection method to select variables based on the Wald *χ*^*2*^ test until all variables remaining in the model were significant (*p* < 0.05). The explanatory variables tested were sex, age, education, yearly household income, occupation, household size, household type, residence, travel history, and season.

## 3. Results

### 3.1. Response rate and sample representativeness

The overall response rate was 93.5% (12,021). Of the 12,021 participants included in the survey, 308 (2.6%) reported having experienced symptoms of gastroenteritis in the previous 4 weeks. The average age was 37.98 years, and the participants tended to reside in households with ≥ 3 persons and in urban areas. They were also more likely to be males (**Table 1**).

**Table 1:** Demographic characteristics and weighted monthly prevalence of reported AGI in the 4 weeks prior to interview in Zhejiang Province, July 2018-June 2019 (n = 12021).

Variable	Proportion of Zhejiang population (%)	Proportion of survey respondents (%)	Monthly Prevalence of AGI		*P*
			%	(95%CI)	
Sex					0.751
Male	51.4	47.4	1.8	(1.4–2.1)	
Female	48.6	52.6	2.0	(1.5–2.2)	
Age(years)					0.554
0–4	1.7	4.5	1.5	(0.5–2.5)	
5–14	4.4	8.7	1.7	(0.9–2.5)	
15–24	3.4	15.7	1.7	(1.1–2.3)	
25–44	15.2	36.0	1.7	(1.3–2.1)	
45–64	40.7	25.8	2.2	(1.7–2.7)	
≥65	34.6	9.3	2.0	(1.2–2.9)	
Education					0.246
Preschool children	5.4	8.3	1.9	(1.1–2.7)	
Illiterate	6.2	6.1	2.0	(1.0–3.1)	
Primary	28.8	22.7	2.1	(1.6–2.6)	
Secondary	36.7	29.3	2.1	(1.6–2.6)	
Tertiary	13.6	16.5	1.3	(0.8–1.8)	
University	9.3	17.1	1.6	(1.1–2.1)	
Yearly household income (CNY)					0.009
0–19999	NA	30.4	2.2	(1.6–2.7)	
20000–49999	NA	39.4	2.2	(1.8–2.7)	
≥ 50000	NA	16.6	1.4	(1.0–1.9)	
No response ^a^		13.6			
Occupation					0.019
Too young to work (including students)	NA	22.1	1.5	(1.0–2.0)	
Housework	NA	17.7	2.3	(1.6–2.9)	
Unemployed	NA	1.7	4.3	(1.5–7.1)	
Retired	NA	2.7	1.2	(0.0–2.4)	
Administrator/Director	NA	1.5	1.2	(0.0–2.8)	
Professional	NA	9.4	1.7	(0.9–2.4)	
Office staff	NA	2.5	2.6	(0.8–4.4)	
Services	NA	11.4	1.9	(1.2–2.6)	
Laborer/ Farmer	NA	21.0	2.3	(1.7–2.8)	
Others	NA	10.0	1.0	(0.4–1.6)	
Household size (no. of persons)					0.136
1–2	38.9	23.2	2.2	(1.6–2.6)	
≥3	61.1	76.8	1.8	(1.5–2.0)	
Household type					0.044
No resident<18 years	NA	48.6	2.1	(1.7–2.4)	
At least one resident<18 years	NA	51.4	1.6	(1.3–1.9)	
Residence					0.878
Urban	68.9	54.4	1.8	(1.4–2.1)	
Rural	31.1	45.6	1.9	(1.5–2.2)	
Travel history					0.291
Yes	NA	4.1	2.1	(0.6–3.5)	
No	NA	95.9	2.0	(1.7–2.2)	
Season					0.001
Spring	NA	24.8	1.5	(1.3–2.2)	
Summer	NA	25.6	3.2	(2.6–3.8)	
Autumn	NA	24.9	1.7	(1.1–2.0)	
Winter	NA	24.7	0.9	(0.5–1.2)	
Area					0.003
Wenzhou	16.1	21.9	1.1	(0.7–1.6)	
Jiaxing	8.0	21.3	2.3	(1.7–2.9)	
Jinhua	9.5	22.6	1.5	(1.0–2.0)	
Quzhou	3.8	20.0	2.3	(1.7–2.9)	
Zhoushan	2.0	14.2	2.1	(1.4–2.7)	

Note. Confidence interval-CI, Not available-NA, Chinese yuan-CNY, ^a^ Individuals who did not respond were excluded from the analysis.

### 3.2 Magnitude and distribution of AGI

The demographic characteristics and descriptions of the people who reported AGI are presented in **Table 1**. Among the respondents, 85 (0.7%) had non-infectious causes and were included in the non-case category, leaving 223 (1.9%) respondents categorized as cases. After excluding these respondents, the overall prevalence of AGI 28 days before the survey, adjusted for age and sex, was 1.8% (95% confidence interval (CI) 1.6–2.1). This represents an average of 0.24 (95% CI 0.21–0.28) episodes of AGI per person-year. A total of 11 people had symptoms of AGI at the time of the interview, and 5.9% (13/223) reported respiratory symptoms, including nasal congestion, sneezing, runny nose, coughing, sputum, sore throat, and otitis. Thus, according to the sixth national census of Zhejiang’s population (54.43 million), an estimated 13.06 million cases of AGI occur in Zhejiang Province annually.

**Fig 2:**
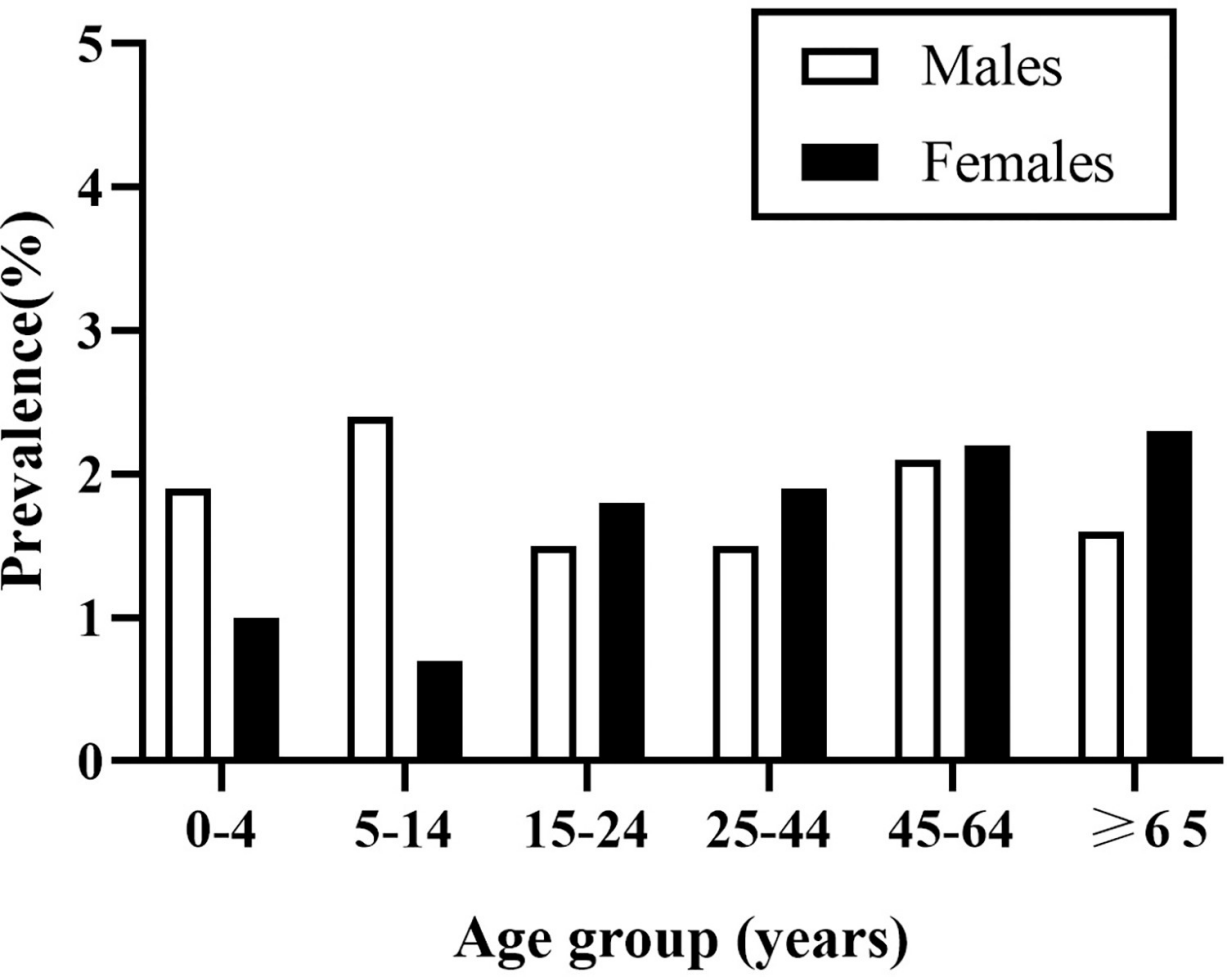
Monthly prevalence of acute gastrointestinal illness by age and sex in the 4 weeks prior to interview in Zhejiang Province, southeast China, July 2018-June 2019.

**Fig 3:**
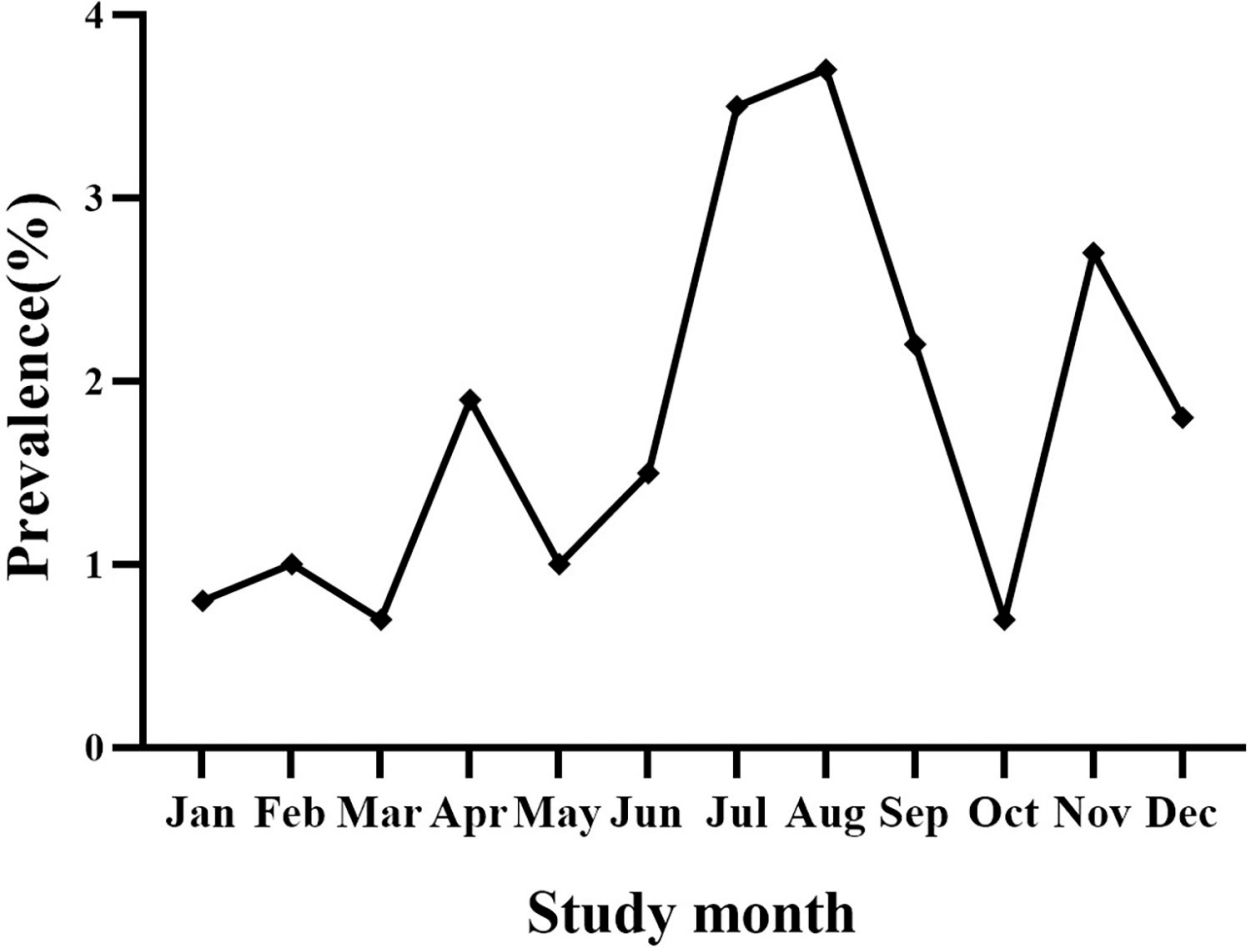
Monthly prevalence of acute gastrointestinal illness by month in the 4 weeks prior to interview in Zhejiang Province, southeast China, July 2018–June 2019.

The estimated monthly prevalence of AGI according to the demographic characteristics is reported in **Table 1**. The prevalence of AGI did not differ significantly according to sex (1.8% *vs*. 2.0%), age (**Fig 2**), education, household size, residence, or travel history. The prevalence of AGI was higher in households with incomes lower than 50,000 CNY (*p* = 0.009), in unemployed people (*p* = 0.019), and in respondents living in households with no residents aged < 18 years (*p* = 0.044). The AGI prevalence was highest in summer (3.2%) (*p* = 0.001) (**Fig 3**). Prevalence varied by sentinel site, with the highest prevalence observed in Jiaxing (2.3%) and Quzhou (2.3%), and the lowest in Wenzhou (1.1%) (*p* = 0.003).

### 3.3. Univariate analysis and multivariable analyses

Univariate analysis showed that the prevalence of AGI was associated with yearly household income, occupation, household type, season, and area (*p* < 0.05) (**Table 2**). Multivariable logistic regression analysis showed that occupation, season, and area were correlated with reported AGI (*p* < 0.05) (**Table 3**). Houseworkers and unemployed individuals were more likely to report an AGI. The prevalence of AGI was higher in summer and autumn than in winter. Respondents who lived in Jiaxing and Quzhou were more likely to report AGI than those who lived at other sites.

**Table 2:** Univariate analysis of the relationship between demographic characteristics and acute gastrointestinal illness in the 4 weeks prior to interview in Zhejiang Province, July 2018-June 2019.

Variable	Univariate analysis		*P*
	OR	(95%CI)	
Sex Male Female			
	Ref.	Ref.	Ref.
	1.138	0.887–1.475	0.331
Age(years) 0–4			
	Ref.	Ref.	Ref.
5–14	1.034	0.271–3.935	0.961
15–24	1.149	0.294–4.492	0.841
25–44	1.147	0.347–3.785	0.822
45–64	1.477	0.465–4.692	0.509
≥65	1.308	0.410–4.179	0.650
Education			
Preschool children Illiterate Primary Secondary	1.113	0.625–1.982	0.717
	1.152	0.685–1.937	0.593
	1.038	0.606–1.777	0.893
	Ref.	Ref.	Ref.
Tertiary University	0.809	0.408–1.607	0.546
	0.728	0.340–1.558	0.413
Yearly household income (CNY)			
0–19999	1.483	0.988–2.226	0.057
20000–49999	1.482	1.016–2.161	0.041
≥ 50000	Ref.	Ref.	Ref.
No response ^a^			
Occupation			
Too young to work (including students)	Ref.	Ref.	Ref.
Housework	1.568	1.017–2.418	0.042
Unemployed	3.039	1.433–6.444	0.004
Retired	0.803	0.274–2.354	0.689
Administrator/Director	0.630	0.124–3.210	0.578
Professional	1.172	0.670–2.047	0.578
Office staff	1.816	0.830–3.973	0.135
Services	1.360	0.823–2.249	0.230
Laborer/ Farmer	1.568	1.033–2.379	0.035
Others	0.671	0.345–1.306	0.240
Household size (no. of persons)			
1–2	Ref.	Ref.	Ref.
≥3	0.811	0.600–1.097	0.173
Household type			
No resident<18 years	Ref.	Ref.	Ref.
At least one resident<18 years	0.759	0.580–0.994	0.045
Residence			
Urban	Ref.	Ref.	Ref.
Rural	1.126	0.835–1.517	0.437
Travel history			
Yes	0.672	0.299–1.512	0.337
No	Ref.	Ref.	Ref.
Season			
Spring	1.702	1.040–2.785	0.034
Summer	3.798	2.451–5.885	< 0.01
Autumn	2.063	1.279–3.325	0.003
Winter	Ref.	Ref.	Ref.
Area			
Wenzhou	Ref.	Ref.	Ref.
Jiaxing	2.142	1.366–3.358	< 0.01
Jinhua	1.389	0.859–2.245	0.180
Quzhou	2.110	1.338–3.328	0.001
Zhoushan	1.913	1.164–3.144	0.011

Note. Confidence interval-CI, Not available-NA, Chinese yuan-CNY. ^a^ Individuals who did not respond were excluded from the analysis.

**Table 3:** Multivariable analysis of yearly household income, occupation, household type, season, area and AGI prevalence in the 4 weeks prior to interview in Zhejiang Province, July 2018-June 2019.

Variable	β	Wald *χ*^2^	OR (95% CI)	*P*
Yearly household income (CNY) 0–19999 20000–49999				
	0.265	1.471	1.303 (0.849–2.000)	0.225
	0.254	1.611	1.290 (0.871–1.910)	0.204
≥ 50000 No response ^a^	-	-	-	-
			
Occupation				
Too young to work (including students)	-	-	-	-
Housework	0.562	4.827	1.754 (1.062–2.895)	0.028
Unemployed	1.189	8.667	3.285 (1.488–7.252)	0.003
Retired	0.448	0.489	0.639 (0.182–2.242)	0.484
Administrator/Director	0.349	0.172	0.706 (0.136–3.664)	0.678
Professional Office staff Services Laborer/ Farmer	0.408	1.773	1.504 (0.825–2.741)	0.183
	0.613	2.176	1.845 (0.818–4.165)	0.140
	0.390	1.845	1.476 (0.842–2.590)	0.174
	0.326	1.769	1.385 (0.857–2.239)	0.183
Others Household type	0.422	1.218	0.656 (0.310–1.388)	0.270
			
No resident<18 years	-	-	-	-
At least one resident<18 years	0.152	0.939	0.859 (0.631–1.169)	0.333
Season				
Spring	0.467	3.162	1.596 (0.953–2.672)	0.075
Summer	1.279	30.192	3.592 (2.276–5.668)	< 0.01
Autumn	0.686	7.316	1.985 (1.208–3.263)	0.007
Winter	-	-	-	-
Area				
Wenzhou	-	-	-	-
Jiaxing	0.907	13.175	2.476 (1.518–4.040)	< 0.01
Jinhua	0.337	1.466	1.401 (0.812–4.040)	0.226
Quzhou	0.756	9.123	2.129 (1.304–3.478)	0.003
Zhoushan	0.484	3.046	1.622 (0.942–2.791)	0.081

Note. Confidence interval-CI, Chinese yuan-CNY. ^a^ Individuals who did not respond were excluded from the analysis.

### 3.4. Symptoms and severity

Among the 223 AGI cases, 216 (96.8%) reported diarrhea and 18 (8.0%) reported vomiting. Other symptoms included nausea (40, 17.9%), abdominal pain (126, 56.4%), loss of appetite (55, 24.6%), fever (10, 4.5%), headache (10,4.5%), muscle pain (8, 3.4%), joint pain (6, 2.8%), otitis (1, 0.3%), and respiratory system symptoms (13, 5.9%).

Among the 233 AGI cases, the mean illness duration was 1.51 days (range 1 hour –30 days), with a median duration of 1.13 days. The duration of illness did not differ significantly between age groups (*p* > 0.05, ANOVA). On the worst day of symptoms, cases reported an average of 4.15 (range: 3–12) events of loose stools and 3.095 (range: 1–9) events of vomiting.

### 3.5. Suspected causes of illness

Patients with AGI were asked to report the most likely causes of their illness. Among the AGI cases, 137 (58.3%) were attributed to food poisoning, while 65 (27.7%) were unknown. Among those with a suspected etiology of contaminated food, the self-reported reasons included aquatic animals (32, 20.5%), vegetables (28, 17.9%), fruits (19, 12.7%), poultry products (15, 9.6%), and animal products (12, 7.7%). Among these cases, the highest proportion of contaminated foods (91, 66.2%) came from their families.

### 3.6. Health care and impact

Of the 233 AGI cases, 231 (99.1%) had medical insurance. Health care-seeking behavior, medication use, and source of the medication are shown in **Table 4**. A total of 130 patients (55.8%) visited a doctor. After adjusting for sex, age, and residence, the prevalence of seeking medical care was 70.2% (95% CI 63.7–76.7). Only three patients were required to provide stool samples for pathogen detection.

**Table 4:** Hospital visit and medicine use by participants with acute gastrointestinal illness in the 4 weeks prior to interview in Zhejiang Province, July 2018-June 2019.

Variable	No. of case (%)
Visit a doctor (n = 233) Yes No	
	130 (55.8)
	103 (44.2)
Submit a stool sample for pathogen testing (n = 88) Yes	
	3 (3.4)
No	85 (96.6)
Take medicine (n = 233)	
Yes	131 (56.2)
No	102 (43.8)
Type of medicine (n = 174) ^a^	
Antidiarrheals	72 (41.5)
Antibiotics Paregoric Antipyretics Antacids	62 (35.6)
	2 (1.1)
	1 (0.6)
	2 (1.1)
Other Unknown	27 (15.5)
	8 (4.6)
Location of medicine purchase (n = 141) ^a^	
Pharmacy	31 (22.0)
Hospitals with prescription	63 (44.7)
Family medicine chest	37 (26.2)
Other	10 (7.1)

^a^ Because some participants took more than one type of medication and some visited more than one location, the total percentage may exceed 100%.

In total, 56.2% of the patients took medicine for the treatment of their conditions, with 41.5% taking antidiarrheals and 35.6% taking antibiotics. The major categories of antibiotics used were quinolones (57.6%) and aminoglycosides (18.5%). Of those who took medicine, 44.7% reported that the medicine was obtained from a hospital with a prescription, 26.2% obtained medicine from the family medicine chest, and 22.2% bought medicine from the pharmacy. Rural hospitals and community health service stations were the most frequently visited medical institutions (109, 46.7%), followed by township health centers or community health services centers (22.7%), and county-level hospitals. However, 161 patients (69.1%) did not visit a doctor. The main reasons were "mild symptoms, no need to go to the hospital" (129 participants, 80.1%) and "self-medication"(57 participants, 35.4%).

### 3.7. The economic burden of disease and its impact on work and school

Regarding the economic burden, the AGI-associated direct medical expenses were 379.33 CNY/person-year, including the treatment cost for patients who did not see a doctor (7.66 CNY/person-year), the outpatient treatment fee (124.18 CNY/person-year), and the treatment expenses of hospitalized patients (247.49 CNY/person-year). The total fee for direct non-medical expenses was 304.22 CNY/person-year, including expenses for transportation and room and board incurred by patients, visitors, and escorts (**Table 5**).

**Table 5:** The direct economic burden of AGI in the 4 weeks prior to interview in Zhejiang Province, July 2018-June 2019.

Items of economic burden	Expenses (CNY/person-year)	Person- year	Estimated cost (million CNY)
**Direct medical expenses** Treatment expenses for patients who did not see a doctor Outpatient treatment fee	379.33	1418	665.15
	7.66	959	73.47
	124.18	441	547.45
Treatment expenses of hospitalized patients **Direct non-medical expenses**	247.49	18	44.23
	304.22	1845	221.59
Transportation expenses for patients without medical treatment	0.14	959	1.34
Transportation expenses of patients	16.73	441	73.75
Additional room and board for the patient	32.57	441	143.59
Transportation expenses incurred by visitors	37.5	1	0.25
Extra room and board for visitors	90.91	1	0.61
Transportation expenses incurred by escorts	57.81	2	0.93
Extra room and board for escorts	68.57	2	1.11
**Total**	683.55	3263	886.74

According to the population data of Zhejiang Province, the direct annual medical expenses caused by AGI were 665.15 million CNY, the direct non-medical expenses were 22.59 million CNY, and the total direct economic burden was 886.74 million CNY (approximately 887 million CNY). Thus, based on the proportion of employees in the total population of Zhejiang in 2017 (76.6%), the estimated indirect economic loss caused by labor loss was 88.38 million CNY.

According to the survey, the average number of days of absenteeism or school suspension was 0.17 days and the total number of days of work absence was 0.3 days. According to the Zhejiang Provincial Statistical Information Network in 2017, the average wage of employed personnel was 100,283 CNY/person-year. Thus, the disease burden caused by AGI in Zhejiang Province was approximately 975 million CNY, accounting for 0.02% of Zhejiang’s GDP (5176.826 billion CNY in 2017).

### 3.8 Standard case definition comparison

For the international comparisons, the proposed minimum set of results of this study is outlined in **Table 6**, according to the standard symptom-based case definition for AGI [22].

**Table 6:** Descriptive statistics of acute gastrointestinal illness following the proposed standard case definition of gastrointestinal illness in Zhejiang Province, China, July 2018–June 2019.

Annual incidence per person-year (95% CI)	0.24 (0.21–0.28)
Annual incidence per person-year in males	0.24
Annual incidence per person-year in females	0.27
Mean age of cases (years)	37.98
Mean duration of illness (days)	1.51
Cases with bloody diarrhoea (%)	2.1
Cases who sought medical care (%)	70.2
Cases submitting a stool sample for testing (%)	3.4
Cases with respiratory symptoms (%)	5.9
Cases with symptoms still ongoing at time of at interview (%)	5.9

## 4. Discussion

This is the first estimate of AGI in Zhejiang Province. Our results revealed the high prevalence in this province. The prevalence of AGI in the 28 days after standardization was 1.8% (95% CI, 1.6–2.1), corresponding to an estimated incidence rate of 0.24 episodes per person-year and an estimated 13.06 million cases of AGI annually. The mean duration of AGI was 1.51 days and AGI led to a large economic burden, accounting for 0.02% of Zhejiang’s GDP.

It is difficult to compare AGI rates between studies owing to the use of different case definitions and study designs. In this study, we used a compatible case definition and compared the results to those of other areas, and observed a lower rate than those reported in Jiangsu Province (0.63), Gansu Province (1.16), and six provinces of the nation’s overall findings (0.56) [11,18,19]. However, the rate was higher than that in Beijing (0.15) [25]. Compared to the situation abroad, there were 0.57 AGI episodes per person-year in Canada [12], 1.11 episodes per person-year in New Zealand [26], 0.95 episodes per person-year in Germany [27], 1.08 episodes per person-year in Italy [28], and 1.4 episodes per person-year in Denmark [29].

Zhejiang differs from other provinces due to different exposures based on geographic location, lifestyle, etc. Zhejiang Province is located on the eastern coast of China and has a humid subtropical climate with four distinct seasons. The annual mean temperature ranges from 15.0 to 18.0°C, and the average annual rainfall is 980–2000 mm across cities, the relative humidity exceeds 94%, and the average temperature of the 11 cities is > 30°C [30,31]. Heavy rainfall events, flooding, and droughts lead to increased enteric infections and hepatitis, which may result in an altered distribution of gastrointestinal illness [32]. In addition, as one of the most developed provinces in China, living standards vary widely between cities. Economic development in the population may affect dietary habits and cause water shortages, poor water quality, and inadequate facilities for food storage and preparation.

In this study, occupation, season, and area were correlated with AGI. Houseworkers and unemployed individuals were more likely to report an AGI. The prevalence of AGI was highest in the summer. Several reasons may explain this observation. First, in summer, climate warming and weather changes exacerbate the challenges of nutrition and clean water. Moreover, owing to the increase in rainfall events in summer, enteric infections and inflammatory bowel disease (IBD) rise accordingly [33,34]. In addition, respondents living in Jiaxing and Quzhou were more likely to report AGI compared to respondents at other sites. There are some plausible explanations for the association between location and AGI prevalence. Primarily, National Health Interview Survey (NHIS) data showed that gastrointestinal illness was associated with income, with a negative correlation between income and overall health, including AGI [35,36]. This may be due to the different lifestyle behaviors in this group, such as health habits, patterns of dining out, and awareness of hygienic conditions [37,38]. Moreover, intestinal microorganisms may have a prominent effect on disease [39,40]. People in different regions have multiple dietary habits (ketogenic, high sugar, Western-type, high salt, Mediterranean diets, etc.), which are associated with AGI due to their diverse gut microbiota compositions [41,42].

Interestingly, more than half of the AGI cases in this study were attributed to contaminated foods. Patients with AGI rarely know the actual cause and often attribute it to food. However, no better method exists to estimate the proportion of foodborne AGIs. Generally, meat, fish, vegetables, fruits, grains, dairy, and aquatic products are contaminated by pathogenic microorganisms [43–47]. The most common pathogenic microorganisms associated with contaminated food are *Salmonella*, *Listeria*, *Colibacillus*, and *Vibrio*, while the nonpathogenic bacteria include *Enterobacteriaceae*, *Salmonella*, and coliforms [48]. Patients with these pathogenic microorganisms present similar clinical symptoms, including nausea, emesis, stomachache, and diarrhea [49,50]. Surprisingly, many patients seem to ignore these consequences. AGI is usually treated with immediate hydration, and, in many cases, does not require any medication, while maintenance intravenous fluids (IVFs) provide vital support for children [51].

Our results revealed that 56.2% of patients with AGI took medicine for the treatment of their conditions, with 41.5% taking antidiarrheals and 35.6% taking antibiotics. The proportion of antibiotic use was higher than that in Hong Kong [52]. Antibiotics are rarely used in the treatment of AGI, with usage rates reported in foreign studies of only approximately 10%. The results of this study indicated the need for further standardization of drug treatments for AGI to avoid drug abuse.

Disease burden is divided into epidemiological and economic burdens. The former refers to the morbidity, hospitalization, disability, and death caused by disease, such as morbidity and hospitalization rates. The latter includes economic loss to patients, families, and society caused by illness, disability, and premature death caused by disease and is usually measured in monetary terms [14,53]. Moreover, the economic cost commonly includes direct loss, which refers to the basis of health care usage and resources, and indirect loss, which is the loss of labor [54,55].

The results of this study revealed relatively large economic losses in Zhejiang Province due to AGI. There are several possible reasons for the high economic burden. First, owing to better regional economic conditions and the developed medical service system, people are more concerned about their own health [56]. Second, in this study, 79.1% of cases were aged > 45 years and were mainly elderly people, which was related to the fact that more basic diseases were more likely to lead to higher treatment costs [57]. Third, the utilization rate of antibiotics in China accounts for half of the rate worldwide, and the use in Zhejiang Province is relatively high [58]. Improper use of antibiotics not only increases the economic burden on patients but also increases bacterial resistance [59]. Antibiotic use can also lead to the disruption of the normal flora balance, endogenous infection, and other adverse drug reactions due to drug allergies, resulting in large social and economic burdens [60]. Therefore, the use of antibiotics should be further investigated.

There are still some shortcomings in our study. First, recall bias is inevitable in retrospective studies and may result in an inaccurate estimate of the true prevalence of AGI. We did not confirm the accuracy of the information provided by respondents, such as duration. To reduce this bias, we attempted to collect data on the actual onset, which yielded more accurate results. Second, the elderly population accounted for a large proportion of the survey, while children and students accounted for only 28.9% of the respondents. We defined AGI cases according to symptoms based on self-reports rather than pathogen-specific laboratory confirmation. The number of stool samples examined for pathogen detection was so small that we could not determine the real causes of the cases. Thus, further laboratory confirmation is required to measure the specific incidence and suspected causes, such as intestinal parasites. However, the highlight of this study is the high response rate compared to previous surveys in other countries. We employed face-to-face interviews and conducted surveys with trained health workers.

## 5. Conclusions

In summary, we concluded that acute gastrointestinal illness (AGI) causes a substantial health burden in Zhejiang Province in southeast China. Further research is needed on the pathogen-specific burden of AGI, as well as efforts aimed at the further investigation and development to reduce the incidence of high-risk mass gastroenteritis.

## Supporting information

S1 File Study questionnaire. (DOC)

S1 Data (XLS)
