# Supplementary material for: A population-based survey of the prevalence of self-reported acute gastrointestinal illness in Zhejiang Province, China
Source: PLoS One. 2022 May 18;17(5):e0268717. doi: 10.1371/journal.pone.0268717 (PMC9116671; doi:10.1371/journal.pone.0268717)
Supplement: S1 File — (DOC) [file pone.0268717.s001.doc]

**Additional file 1:** **Study questionnaire.**

T1. Date of interview Years/Months/Days

F2. Person code ________________

F3. Current Home Address

City F3a________

District/County F3b ________

Street/Town F3c ____________

Committee/Village F3d ___________

Number F3e______________

F4. Residence [1] Urban [2] Rural

F5. Name of interviewer ______________ F6 Name of assessor _____________

**Section one: Basic information**

N1. How many people are usually live in this household? ______

N1.1 How many are <18 years of age? ______ (enter ‘0’ if none)

N2. Name of family member whose birthday is coming ______

N3. If the questionnaire being answered by the selected respondent him or herself?

[1] Yes (skip to N4) [2] No

N3.1. If not answered by the respondents themselves, the name of the respondents ______

N3.2. You are the subject of ______ (For example: father, mother, etc)

N4. Telephone ____________

N5. Gender [1] Male [2] Female

N6. Date of birth (refer to ID card) ____________Years/Months/Days

N7. Ethnic group [1] Han [2] National minority

N7.1 If ethnic minority, please specify______

N8. Education

[1] Preschool children [2] Illiterate [3] Primary school [4] Secondary school

[5] High school [6] Technical secondary school and junior college

[7] University [8] Postgraduate

N9. Occupation

[1] Child in child care settings [2] Child outside child care settings [3] Student

[4] Housekeeper [5] Unemployed [6] Retired

[7] Administrator and office staff Service personnel [8] Professionals

[9] Clerical and related personnel [10] Business service personnel

[11] Agriculture, forestry, animal husbandry, fishery and water conservancy

[12] Production and transportation equipment operators [13] Soldier [14] Other

N10. During the past two weeks did you travel outside of the district/county where your

household resides? [1] Yes [2] No (go to N11)

**The past 2 weeks, that is from (date)** ______**through (date)** ______**.**

N10.1 Place name (______Province ______Prefecture)

N11. Total family income per year ___________ yuan

[1] 5000 yuan of the following [2] 5000-9999 yuan [3] 10000-14999 yuan

[4] 15000-19999 yuan [5] 20000-24999 yuan [6] 25000-29999 yuan

[7] 30000-34999 yuan [8] 35000-39999 yuan [9] 40000-44999 yuan

[10]45000-49999 yuan [11] 50000-54999 yuan [12] 55000-64999 yuan

[13] 65000-79999 yuan [14] More than 80000 [0] No answer

**Section two: Clinical Symptoms**

In the past four weeks, taking the survey date of July 29 as an example, the following should be the last four weeks

①Symptoms of gastroenteritis developed between July 1 and July 28;

②Symptoms of gastroenteritis appeared before July 1, but the course lasted from July 1 to July 28.

**The past 4 weeks, that is from (date)** ______ **through (date)** ______**.**

N12. During the past 4 weeks, have you suffered from diarrhea or vomiting? Diarrhoea

was ≥ 3 loose stools in a 24-hour period. [1] Yes [2] No (go to N14)

N13. Whether you were diagnosed by a doctor (a) with intestinal cancer, inflammatory

bowel disease like Crohn’s disease and ulcerative colitis, acute pancreatitis, irritable

bowel syndrome, colitis, diverticulitis or other chronic disease with symptoms of diarrhea

or vomiting, or pregnancy, excessive alcohol consumption, chemotherapy/radiotherapy,

medication, menstruation, food allergies? [1] Yes [2] No

N13.1. If yes, which illness? _____________________

N14. Judged by the interviewer, how many episodes of acute gastrointestinal illness did the respondent have during the past 4 weeks? ____ [(1) enter ‘0’ if N14= 2; (2) enter ‘0’ if n13=1; (3) enter ‘≥2’if a 7-day symptom-free interval was defined to distinguish multiple episodes]

**If N14 = 0, go to the place of signature.**

If N14 ≥ 1, respondents were asked to respond only for their most recent episode for the remaining questions.

N15. Have you suffered from loose stools? [1] Yes [2] No (go to N16)

N15.1. If yes, how many times in a 24-hour period? ____

N15.2. Have you suffered from bloody diarrhea? [1] Yes [2] No (go to N16)

N15.2.1. If yes, how much blood was there in your stool? ___

[1] Just a little blood on the toilet paper

[2] Some blood mixed with the stool

[3] So much blood that the stool was almost entirely blood

N16. Have you suffered from vomiting? [1] Yes [2] No (go to N17)

N16.1 If yes, how many times in a 24-hour period? ___

N17. Have you also experienced the following symptoms?

N17.1. Nausea [1] Yes [2] No

N17.2. Abdominal pain [1] Yes [2] No

N17.3. Inappetence [1] Yes [2] No

N17.4. Fever [1] Yes [2] No

N17.5. Headache [1] Yes [2] No

N17.6. Muscle pain [1] Yes [2] No

N17.7. Joint pain [1] Yes [2] No

N17.8 Otitis [1] Yes [2] No

N17.9. Others, please specify ______________ (symptoms here except those belong to respiratory system)

N18. Have you experienced the following respiratory system symptoms? [1] Yes [2] No (go to N19)

N18.1. Nasal congestion [1] Yes [2] No (go to N23)

N18.2. Sneezing [1] Yes [2] No

N18.3. Runny nose [1] Yes [2] No

N18.4. Coughing [1] Yes [2] No

N18.5. Sputum [1] Yes [2] No

N18.6. Sore throat [1] Yes [2] No

N18.7. Others, please specify ______________

N19. Are you still suffering from symptoms of acute gastrointestinal illness today? [1] Yes (go to N21) [2] No

N20. How long did the illness last? Days _____ Hours ____

N21. In your opinion, what do you think was the most possible cause of your illness? (If you select 2, 3, 4, or 6, go to N24)

[1] Food poisoning [2] Person-to-person [3] Contaminated water

[4] Animal contact [5] Other [6] Unknown

N21.1 Other, please specify _______

**Section three: Suspected food**

N22. If food poisoning, which food you think was most suspected to cause your symptoms? ______

N22.1. Type of food

[1] Cereals and cereal products [2] Potato, starch and products

[3] Beans and bean products [4] Vegetable products [5] Thallophyte

[6] Fruits and fruit products [7] Nuts and seeds [8] Livestock meat and products

[9] Poultry meat and products [10] Milk and dairy products

[11] Eggs and egg products [12] Fish, shrimp, crab and shellfish

[13] Infant food [14] Snack and dessert [15] Fast food [16] Drink

[17] Alcoholic drink [18] Sugar, preserved and preserved fruit, honey

[19] Oil [20] Condiments [21] Other

N23. If food poisoning, where do you think you got the food that caused your symptoms?

[1] Own home [2] Private house (excluding own home) [3] Hotel/Restaurant

[4] Fast food service [5] Food supermarket [6] Delicatessens [7] Bakery

[8] Drinks shop [9] Street vendor [10] Takeaway

[11] School cafeteria [12] Company cafeteria

[13] Food service on construction sites

[14] Farmer’s market [15] Other [16] Unknown

N23.1. Other, please specify___________________

**Section four: Medical treatment**

N24. Did you have medical insurance? [1] Yes [2] No (go to N25)

Type of medical insurance (multiple options)

N24.1 Basic medical insurance system for urban worker [1] Yes [2] No

N24.2 Basic medical insurance system for urban residents [1] Yes [2] No

N24.3 Free medical service [1] Yes [2] No

N24.4 Labor-protection medical care [1] Yes [2] No

N24.5 The New Rural Cooperative Medical Care System (NRCMCS)

N24.6 Commercial health insurance

N24.7 Other ________________

N25. Did you go to the hospital because of illness? [1] Yes (go to N27) [2] No

N26.Reason for not seeing a doctor (multiple choices)

N26.1. Did not think it was severe enough to seek medical care [1] Yes [2] No

N26.2. Self-medication [1] Yes [2] No

N26.3. Too busy [1] Yes [2] No

N26.4. The expenditure for seeking healthcare is high [1] Yes [2] No

N26.5. Transportation problems [1] Yes [2] No

N26.6. Have no medical insurance [1] Yes [2] No

N26.7. Distrust doctors [1] Yes [2] No

N26.8. The healthcare environment is not good enough [1] Yes [2] No

(N26 answer end, skip to N32)

N27. How many _____days_____ hours after your illness, you have sought medical care?

(For example: 8 hours, filled in 0 days 8 hours; 2 days, filled in 2 days 0 hours)

N28. Name of first medical institute _________________

N28.1. Grade of hospital

[1] Tertiary hospital [2] Secondary hospital [3] Class-Ⅰ hospital [4] Other

N28.2. Character of hospital

[1] General hospital [2] Children’s hospital

[3] Traditional Chinese medicine hospital [4] Other

N28.3. Type of hospital

[1] Provincial and ministerial hospitals [2] Prefecture-level hospitals

[3] County-level hospitals

[4] Township health center or Center of Community Health Service

[5] Rural hospital or community health service station [6] Private hospital

[7] Individual clinic [8] Other

N29. As a result of this illness whether you hospitalized? [1] Yes [2] No (go to N31)

N29.1. If yes, how many _____days_____ hours were you hospitalized?

(For example: 8 hours, filled in 0 days 8 hours; 2 days, filled in 2 days 0 hours)

N30. Name of hospital you admitted in firstly ___________________________

N30.1. Grade of hospital

[1] Tertiary hospital [2] Secondary hospital [3] Class-Ⅰ hospital [4] Other

N30.2. Character of hospital

[1] General hospital [2] Children’s hospital

[3] Traditional Chinese medicine hospital [4] Other

N30.3. Type of hospital

[1] Provincial and ministerial hospitals [2] Prefecture-level hospitals

[3] County-level hospitals

[4] Township health center or Center of Community Health Service

[5] Rural hospital or community health service station [6] Private hospital

[7] Individual clinic [8] Other

N31. Were you asked to submit a stool sample for pathogen testing? (not including routine tests alone) [1] Yes [2] No (go to N32)

N31.1. Lab test results ___________ (enter the aetiology being identified by the laboratory, if not sure, enter ‘unknown’)

N32. Did you take any medications for this illness? [1] Yes [2] No (go to N35)

N32.1. Pharmacies buy their own drugs [1] Yes [2] No

N32.2. Hospitals with prescription [1] Yes [2] No

N32.3. Family medicine chest [1] Yes [2] No

N32.4. Other, please specify ___________________

N33. How many _____days_____ hours were medications taken for?

(For example: 8 hours, filled in 0 days 8 hours; 2 days, filled in 2 days 0 hours)

N34. Name of the medication(s) ______ (can fill a variety of drugs, enter ‘unknown’ if not sure)

Type of medicine

N34.1. Antidiarrheals [1] Yes [2] No

N34.2. Antibiotics [1] Yes [2] No (go to N34.3)

N34.2.1. Antibiotics name ______ (can fill a variety of drugs, enter ‘unknown’ if not sure)

N34.2.2. Class of antibiotics (Multiple drugs of optional)

N34.2.2a Pencillins [1] Yes [2] No

N34.2.2b Cephalosporins [1] Yes [2] No

N34.2.2c Carbapenems [1] Yes [2] No

N34.2.2d Polypeptide [1] Yes [2] No

N34.2.2e Chloramphenicols [1] Yes [2] No

N34.2.2f Lincomycin [1] Yes [2] No

N34.2.2g Aminoglycosides [1] Yes [2] No

N34.2.2h Quinolones [1] Yes [2] No

N34.2.2i Macrolides [1] Yes [2] No

N34.2.2j Tetracyclines [1] Yes [2] No

N34.2.2k Folate metabolism inhibitors [1] Yes [2] No

N34.2.2l Other [1] Yes [2] No

N34.3. Paregoric [1] Yes [2] No

N34.4. Antipyretics [1] Yes [2] No

N34.5. Antacids [1] Yes [2] No

N34.6. Other [1] Yes [2] No

N34.7. Unknown [1] Yes [2] No

**Section five: Social and economic impact of illness**

N35. Cases

N35.1. Cost of medication by case who did not visit a doctor _______ yuan (enter ‘0’ if none)

N35.2. Travel cost by case who did not visit a doctor _______ yuan (enter ‘0’ if none)

(The unattended patient jumps to N37 after answering N35)

**Note: If you have taken medicine before the visit, you need to fill in N35 and N36.**

N36. Patient (enter ‘0 ’if none)

N36.1. Cost for out-patient treatment (Total cost. Including all kinds of medical insurance payment) _______ yuan (enter ‘0 ’if none)

N36.2. Cost for inpatient treatment (Total cost. Including all kinds of medical insurance payment) _______ yuan

N36.3. Travel cost for medical care (Total cost. Including all kinds of medical insurance payment) _______ yuan (enter ‘0’ if none)

N36.4. Additional cost of food and accommodation (Total cost. Including all kinds of medical insurance payment) _______ yuan (enter ‘0’ if none)

N37. Did this illness require you to miss work or school/college?

[1] Yes [2] No (go to N38)

N37.1. Missed from work days hours (Absence from work/class, limit to one item)

N37.2. Missed from school/college days hours (Absence from work/class, limit to one item)

(For example: 8 hours, filled in 0 days 8 hours; 2 days, filled in 2 days 0 hours)

N38. Visitors (enter ‘0’ if none)

N38.1. How many times did someone visit you while you were in the hospital? _______ (enter ‘0’ if none, and go to N40)

N38.2. Travel cost _______yuan

N38.3. Additional cost of food and accommodation _______ yuan

N38.4. Missed from work by visitors days hours

N39. Caregivers

N39.1. No. of caregivers _______ person-time (enter ‘0’ if none, and go to N41)

N39.2. Travel cost _______ yuan (enter ‘0’ if none)

N39.3. Additional cost of food and accommodation _______ yuan (enter ‘0’ if none)

N39.4. No. of days missed from work by caregivers days hours

(For example: 8 hours, filled in 0 days 8 hours; 2 days, filled in 2 days 0 hours)

N39.5. Cost of paid help _______ yuan (enter ‘0’ if none)

N40. Did anyone else in your household have suffered from acute gastrointestinal illness in the past 4 weeks? If any, how many? _______ (enter ‘0’ if none)
